# A Porous Chitosan Film-Based Dual-Modal Biosensor Integrated with Catalytic Hairpin Assembly for Sensitive Detection of *Escherichia coli*

**DOI:** 10.3390/bios16100556

**Published:** 2026-10-02

**Authors:** Yuhuan Wei, Qibin Wu, Hui Zhang, Jiayao Lin, Zhen Su, Sirui Yi, Kang Yang, Xiaomeng Jia, Shanwen Hu

**Affiliations:** 1 Department of Health Inspection and Quarantine, School of Public Health, Fujian Medical University, Fuzhou 350122, China; 2260220301@fjmu.edu.cn (Y.W.); wqb2250220260@fjmu.edu.cn (Q.W.); zzzh264118@fjmu.edu.cn (H.Z.); 3240333044@stu.fjmu.edu.cn (J.L.); suzhen@stu.fjmu.edu.cn (Z.S.); ysr165@fjmu.edu.cn (S.Y.); 3240333050@stu.fjmu.edu.cn (K.Y.); 2 Key Laboratory of Analysis and Detecting Technology, Food Safety MOE, College of Chemistry, Fuzhou University, Fuzhou 350002, China; 2501320092@fzu.edu.cn

**Keywords:** dual-modal biosensor, catalytic hairpin assembly, *Escherichia coli*

## Abstract

Catalytic hairpin assembly (CHA), as an enzyme-free isothermal nucleic acid amplification technique, offers significant advantages in biosensing owing to its exponential signal gain. Integrating CHA with dual-modal signal output holds promise for further improving the sensitivity and reliability of pathogen detection in complex matrices through cross-validation of multi-dimensional information. However, when two signals are generated within the same reaction system, they are prone to crosstalk and competition, while the interfacial reaction is inherently constrained by steric hindrance and limited mass transfer. To address these issues, we report herein a CHA-based dual-modal detection strategy for *Escherichia coli* nucleic acid. In this design, the electrochemiluminescence (ECL) signal is localized at the electrode interface, whereas the fluorescence signal is released in the bulk solution, thereby circumventing energy competition and channel crosstalk between the two signals. Concurrently, a three-dimensional scaffold is constructed using the porous structure of phase-transformed chitosan, which provides a high specific surface area to enhance the interfacial loading of hairpin probes and offers unobstructed channels for efficient mass transfer of co-reactants and target molecules, thus alleviating steric hindrance and diffusion limitations. Upon recognition of the target *E. coli* nucleic acid, the CHA cascade is triggered, driving a programmed conformational transition of the hairpin probes and simultaneously modulating the fluorescence resonance energy transfer (FRET) between FAM-BHQ pairs as well as the electrochemiluminescence resonance energy transfer (ECL-RET) between CdS quantum dots and AuNPs, enabling synchronous output and mutual validation of the dual-modal signals. Under optimized conditions, the proposed strategy exhibits satisfactory sensitivity, stability, and selectivity. This work provides a feasible approach for the deep integration of CHA amplification with dual-modal sensing, offering a reliable tool for rapid and accurate screening of pathogenic microorganisms.

## 1. Introduction

Pathogenic microorganisms are a class of microbes capable of causing infections in humans and animals and triggering epidemic diseases in populations [1,2,3,4]. Rapid and accurate detection of pathogenic microorganisms is a critical technical support for public health emergency response and food safety assurance. Nucleic acid amplification techniques are methods that amplify target nucleic acid fragments from minute initial templates using specific primers, DNA polymerases, and other reagents, thereby enabling specific nucleic acid detection. With the advancement of molecular biology, various novel isothermal nucleic acid amplification techniques have emerged, such as rolling circle amplification (RCA) and loop-mediated isothermal amplification (LAMP), which are sensitive and efficient. However, impurities in real samples often compromise enzyme activity and reduce amplification efficiency, rendering these methods less convenient for detecting trace amounts of microorganisms [5,6]. The emergence of enzyme-free amplification strategies helps to fundamentally address this issue. Common enzyme-free amplification strategies include hybridization chain reaction (HCR) and catalytic hairpin assembly (CHA). These strategies enable nucleic acid signal amplification without the involvement of enzymes or thermal cyclers, thereby simplifying experimental procedures and reducing assay costs while maintaining the high sensitivity and specificity of nucleic acid detection systems [7]. The core principle of CHA relies on toehold-mediated strand displacement: the target strand triggers the cascade opening of hairpin probes to form thermodynamically stable duplexes, while the target strand is released to participate in subsequent cyclic reactions [8]. Unlike the linear amplification of HCR, CHA achieves exponential signal amplification by releasing short duplexes during hairpin assembly to trigger multiple hybridization cycles, and has been widely employed in the construction of low-cost sensing platforms with various signal types, including fluorescence, electrochemistry, and ECL [9,10]. For instance, Shi et al. [11] combined target-induced four-way DNA junction structures with CHA signal amplification to construct a highly sensitive electrochemical biosensor for detecting trace single nucleotide polymorphisms in hairpin DNA associated with Alzheimer’s disease. Xu and his team [12] developed an isothermal sensing platform based on CHA and DNAzyme amplification, in which the target triggers CHA to generate duplex DNA sequences, activating DNAzyme for sustained substrate cleavage and resulting in fluorescence signal amplification. However, despite significant progress made by existing CHA-based single-signal sensing systems, the single-signal output mode is highly susceptible to environmental fluctuations, electrode interface contamination, or background interference in complex samples, leading to insufficient reliability and sensitivity [13,14].

The construction of biosensors with dual signals represents a highly promising solution. The dual-signal readout mechanism not only provides mutually corroborating quantitative information within a single system, effectively eliminating false positive or false negative results, but also enables self-calibration through signal ratios or synergistic variations [15]. For example, Gil et al. [16] developed an orthogonal biosensing platform integrating surface-enhanced Raman scattering (SERS) and graphene field-effect transistor (GFET) measurements for the detection of target analytes. The optical Raman features and electrical responses provided complementary information through distinct transduction mechanisms, demonstrating the potential of orthogonal dual-modal sensing for obtaining more comprehensive and reliable analytical information. Similarly, Cai et al. [17] developed a dual-mode biosensor integrating colorimetric and surface-enhanced Raman scattering (SERS) signals for the discrimination and detection of bacteria. The colorimetric signal enabled sensitive quantitative detection, while the SERS fingerprints provided additional information for bacterial discrimination, demonstrating the potential of complementary signal modalities for improving the sensitivity and identification capability of bacterial sensing.

Among various signal readout modes, fluorescence detection has been widely employed in sensing due to its operational simplicity and high selectivity. However, its inherent susceptibility to optical background interference from sample matrices limits its quantitative reliability in complex systems [18]. In contrast, electrochemical analysis techniques convert chemical information into detectable electrical signals, enabling qualitative identification and quantitative analysis of target microorganisms through signal acquisition and conversion. Electrochemical methods offer outstanding advantages including near-zero background interference, broad linear ranges, and high sensitivity, making them the preferred tools for trace analysis in complex matrices [19]. Nevertheless, despite its excellent sensitivity, a single electrochemical mode lacks built-in calibration mechanisms to exclude spurious results arising from variations in electrode status or fluctuations in co-reactants. Therefore, the combination of fluorescence and electrochemistry allows the use of electrochemistry as the primary high-sensitivity quantitative signal output, while leveraging the fluorescence signal as an independent validation module, achieving ideal complementarity between high sensitivity and high reliability [20]. In recent years, researchers have attempted to apply fluorescence-electrochemical dual-signal biosensors to pathogen detection. For instance, Gao et al. [21] constructed a dual-mode biosensor by integrating CHA and HCR cascade amplification with the cis- and trans-cleavage activities of CRISPR/Cas12a, demonstrating high sensitivity and selectivity in the detection of *Staphylococcus aureus*. Feng et al. [22] developed a fluorescence-ECL ratiometric sensor utilizing the FRET effect between FAM fluorophores and AuNPs, as well as the ECL-RET response between CdS quantum dots (CdS QDs) and AuNPs, achieving ultrasensitive detection of *Staphylococcus aureus* through the self-calibration feature of inversely varying dual signals. Although the above studies have validated the feasibility of dual-signal strategies, several issues remain to be addressed. On one hand, when two signals are generated within the same system, they are confronted with crosstalk or competition; moreover, when interfacial reactions are involved, the trade-off between probe immobilization density and steric hindrance inevitably arises, along with concomitant limitations in mass transfer efficiency. On the other hand, how to achieve non-interfering synchronous output of two signals, avoiding signal crosstalk caused by overlapping energy transfer pathways or conflicting reaction conditions, remains a technically unresolved challenge.

This study integrates the energy transfer mechanisms between nanomaterials with the CHA reaction to construct a dual-modal pathogen detection platform that simultaneously outputs fluorescence and ECL signals for highly specific and sensitive detection of *E. coli.* The core design of this sensor relies on target-triggered CHA cascade reactions to achieve cyclic self-assembly of hairpin probes and signal amplification. Specifically, the FRET effect is exploited to generate fluorescence signals in the bulk solution, while the synergistic action between CdS QDs and AuNPs produces ECL signals at the electrode interface. Notably, a porous chitosan film is introduced at the ECL interface in this study. On the one hand, its large specific surface area provides high-density immobilization sites for CdS quantum dot-functionalized hairpin probes, significantly increasing the loading of active molecules per unit area. On the other hand, its porous framework offers unobstructed channels for efficient mass transfer of ECL co-reactants and target nucleic acid molecules at the electrode interface, thereby effectively enhancing the initial intensity and response rate of the ECL signal and substantially improving the reliability and stability of the detection. The platform exhibits favorable sensitivity and selectivity, providing a reliable tool for rapid identification and quantitative analysis of pathogenic microorganisms.

## 2. Materials and Methods

### 2.1. Instrumentation and Materials

Sodium citrate and other inorganic reagents were purchased from Shanghai Macklin Biochemical Technology Co., Ltd. (Shanghai, China). Buffers such as 5 × TBE were obtained from Shanghai Beyotime Biotechnology Co., Ltd. (Shanghai, China). All oligonucleotides (sequences listed in Table S1), gel electrophoresis reagents, and DNA extraction kits were provided by Shanghai Sangon Biotech Co., Ltd. (Shanghai, China). Chitosan (CS, Deacetylation 95%, 100-200 mpa.s) and bovine serum albumin (BSA, 98%) were purchased from Shanghai Aladdin Biochemical Technology Company Ltd. (Shanghai, China). Deionized water was used throughout the experiments. All reagents were of analytical grade and used as received without further purification.

Fluorescence signal measurements were performed on an F-4500 fluorescence spectrophotometer (Hitachi, Ltd., Tokyo, Japan). Electrochemical signals were monitored using a CHI660E electrochemical workstation (CH Instruments Co., Ltd., Shanghai, China). ECL signals were recorded on an MPI-E analyzer (Xi’an Remex Analysis Instrument Co., Ltd., Xi’an, China). Transmission electron microscopy (TEM) image was acquired by an HT7700 TEM (Hitachi, Japan). UV-Vis absorption spectra were measured using an Infinite 200 Pro multimode microplate reader (Tecan Trading Co., Ltd., Shanghai, China).

### 2.2. Nanomaterial Synthesis

CdS QDs were synthesized via a classical aqueous reflux method [23]. Briefly, 50 mL of 0.01 mol/L CdCl_2_·1/2H_2_O solution was placed in a pretreated three-neck flask, followed by the addition of 250 μL of 3-mercaptopropionic acid. The mixture was stirred continuously at constant temperature for 30 min under nitrogen flow to remove dissolved oxygen. Subsequently, 1 mol/L NaOH solution was added to adjust the pH to 11, during which the solution turned from colorless and transparent to milky turbid and finally became clear. Then, 5.5 mL of 0.1 mol/L Na_2_S solution was introduced, and the solution turned golden-yellow and clear. The reaction was allowed to proceed under continuous stirring at constant temperature for 4 h. After heating was stopped, the solution was cooled to room temperature under continuous nitrogen purging. Isopropanol was added to the resulting reaction solution, and the precipitate was collected by centrifugation, washed with isopropanol, and then dispersed in ultrapure water to obtain CdS QDs, which were stored at 4 °C in the dark for subsequent use. The morphology and particle size of the QDs were characterized by transmission electron microscopy (TEM). The particle size distribution and zeta potential were determined by dispersing 1 mL of the dispersion in a Malvern analyzer. The UV-vis absorption spectrum was recorded using a micro-volume ultraviolet-visible spectrophotometer.

AuNPs were synthesized by the sodium citrate reduction method [24,25]. The brief procedure was as follows: 500 μL of 1% HAuCl_4_ solution was mixed with 49.5 mL of ultrapure water in a 50 mL centrifuge tube, and the mixture was then transferred to a pretreated three-neck flask. After vigorous stirring under reflux for 5 min, 1.65 mL of 38.8 mmol/L sodium citrate solution was rapidly added. The solution gradually turned from pale yellow to wine red. Heating and stirring were continued for 10 min to ensure complete reaction. After heating was discontinued, the solution was allowed to cool naturally to room temperature and transferred to centrifuge tubes. Then the product was purified by centrifugation, dispersed in ultrapure water, and stored at 4 °C in the dark for further use. The morphology of the synthesized AuNPs was characterized by TEM. The particle size and zeta potential were measured using a Malvern particle size analyzer. The UV-vis absorption spectrum was recorded using a micro-volume ultraviolet-visible spectrophotometer.

### 2.3. Construction of Nanoprobes and Electrode Pretreatment

AuNPs@DNA conjugates were prepared by a stepwise salt-aging method [26]. Briefly, the II-H2 nucleic acid strand (100 μmol/L) was diluted to 200 nmol/L with 10 mmol/L TCEP as the dilution solvent and allowed to stand at room temperature for 1 h to reduce disulfide bonds. The reduced II-H2 was then mixed with AuNPs at a volume ratio of 1:15 and incubated at room temperature for 12 h, followed by gradual addition of NaCl to a final concentration of 200 mmol/L through stepwise salt-aging. The resulting solution was placed at 4 °C for 24 h to obtain more stable AuNPs@DNA conjugates.

CdS@DNA conjugates were prepared via EDC-NHS covalent coupling [27,28]. Briefly, 100 μL of CdS QDs solution was added to 900 μL of MES buffer (pH 6.0) and vortexed to disperse. Subsequently, 6 mg of EDC and 3 mg of NHS were added. The mixture was stirred at room temperature to activate the surface carboxyl groups. Then, 10 μL of 100 μmol/L II-H1 nucleic acid probe was introduced, and the reaction was allowed to proceed under stirring at room temperature in the dark for 48 h to enable covalent coupling between the activated carboxyl groups and the amino groups of the nucleic acid. After the reaction, the supernatant was discarded, and the product was washed with 1 × PBS buffer (pH 7.4) containing 0.15 mol/L NaCl, then redispersed in the same buffer and stored at 4 °C.

The porous chitosan film was fabricated by galvanostatic electrodeposition [29]. Briefly, 50 mg of chitosan was dissolved in 5 mL of water. After acidification to approximately pH 3 with HCl, the solution was filtered to remove insoluble residues and subsequently adjusted to pH 5 with 1 mol/L NaOH. Next, 100 μL of AuNP suspension was added to the chitosan solution, and the mixture was vigorously stirred at room temperature for 10 min to obtain the electrodeposition solution. Then, a glassy carbon electrode (GCE) was sequentially polished with 1.0, 0.3, and 0.05 μm alumina powder on a chamois leather pad to a mirror finish, followed by ultrasonic cleaning in water and absolute ethanol. The cleaned electrode was then electrochemically activated in a three-electrode system using 0.1 mol/L KCl containing 5 mmol/L K_3_[Fe(CN)_6_] as the electrolyte. Cyclic voltammetry was performed over the potential range from −0.2 V to 0.6 V at a scan rate of 50 mV/s until stable and reproducible peak currents were achieved. The pretreated and activated GCE was used as the working cathode, with a platinum wire electrode as the counter electrode, and both were immersed in a prepared chitosan/AuNPs mixed electrodeposition solution. Chronopotentiometry was performed with a constant current density of 20 A m^−2^ applied to the GC electrode for 5 min.

### 2.4. Dual-Signal Detection Based on Fluorescence and Electrochemiluminescence (ECL)

Fluorescence signal detection: In a sterile, nuclease-free 1.5 mL centrifuge tube, the annealed I-H1 solution (400 nmol/L), I-H2 solution (400 nmol/L), and buffer (1 × PBS containing 0.15 mol/L NaCl) were sequentially added. Subsequently, a predetermined concentration of the target molecule was introduced into the probe solution, and the mixture was incubated at 37 °C in a metal bath for 6 h. The fluorescence signal intensity was then recorded by scanning at an excitation wavelength of 495 nm. Each sample was measured in triplicate.

ECL signal detection: The annealed II-H1 solution was added to the EDC/NHS activation solution (3 mg EDC and 1.5 mg NHS dissolved in 1 × PBS) to achieve the desired final concentration. Then, 10 μL of this mixture was dropped onto the modified electrode surface and incubated at 4 °C in a humidified chamber for 24 h. After incubation, the initial ECL signal was measured. Subsequently, the II-H2 solution at the same concentration as II-H1, along with a predetermined concentration of target, was introduced and incubated for 1 h, after which the signal was again recorded. The relative response change was calculated from the ECL signals measured before and after target addition and recorded. The electrode potential was scanned from 0 to −2.45 V, with the photomultiplier tube voltage maintained at 480 V and the scan rate set to 0.15 V/s. Each sample was measured in triplicate.

### 2.5. Sample Detection

Samples including vegetables, water, and soil were collected. Briefly, soil samples were collected from a depth of 0-20 cm, followed by removal of impurities, quartering, and low-temperature storage. Water samples were collected from terminal tap-water points and lake-water sites using sterile containers and stored at low temperature. Vegetable samples were collected from multiple sites, and 5 g of each sample was mixed with 45 mL of sterile physiological saline. The samples were stored at low temperature before DNA extraction. Total DNA was extracted from these samples using a DNA extraction kit. The accuracy of the proposed method was evaluated through spike recovery experiments: different concentrations of *E. coli* O157:H7 standard strains were spiked into blank samples before DNA extraction, followed by incubation under the conditions described for fluorescence signal detection in Section 2.4, and the recoveries as well as the relative standard deviations (RSDs) were determined. Meanwhile, the detection results obtained by the proposed method for the real samples were compared with those measured by qPCR. Pearson correlation analysis was performed to calculate the correlation coefficients between each detection mode and the qPCR results, thereby verifying the accuracy and reliability of the developed detection system.

## 3. Results and Discussion

### 3.1. The Principle of CHA-Based Dual-Modal Detection

**Figure 1:**
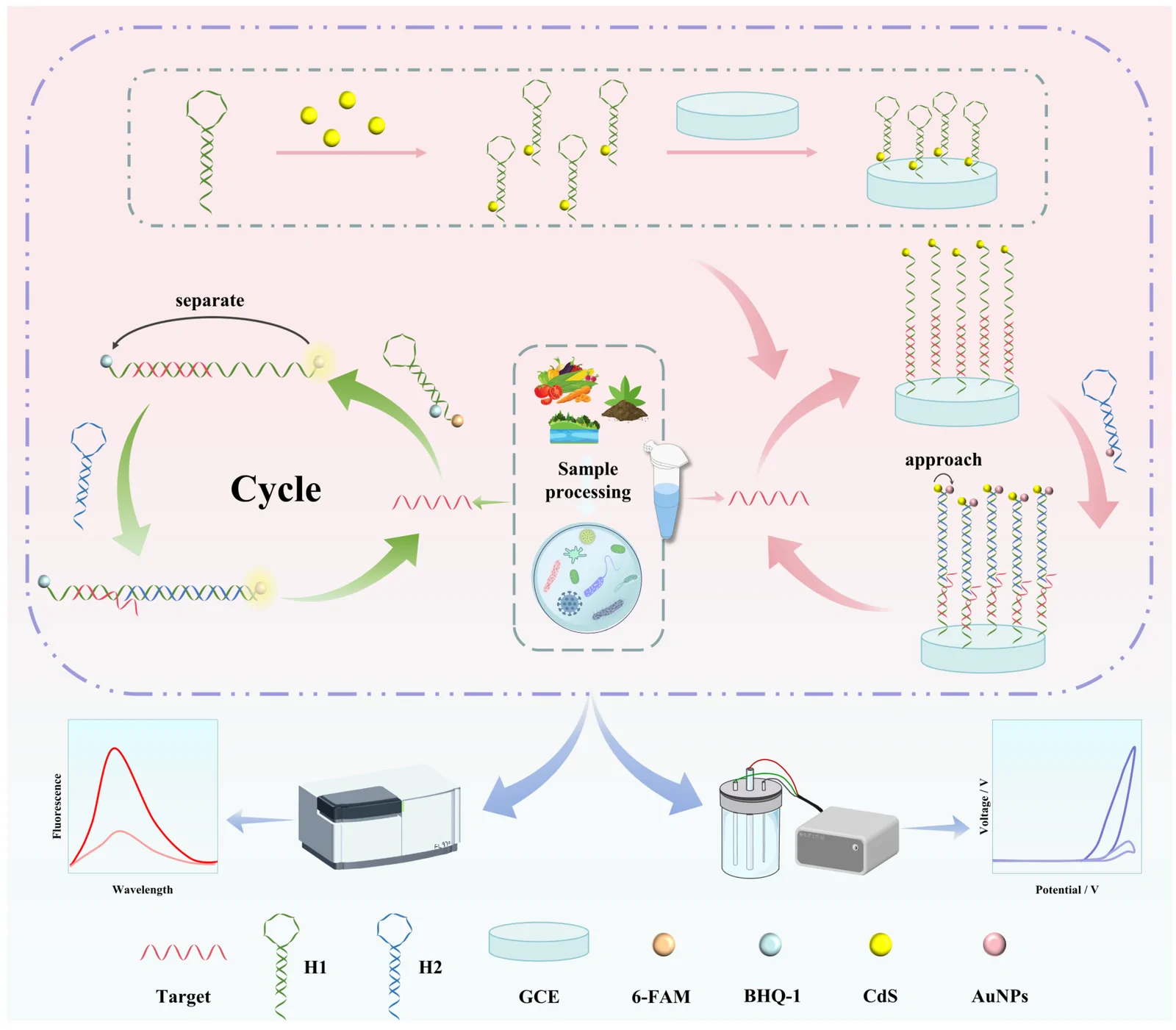
Illustration of a CHA-based dual-modal biosensor.

This study constructed a dual-modal pathogen detection platform based on the CHA technology established by Yin et al. [30], which simultaneously outputs fluorescence and ECL signals for highly specific and sensitive detection of *E. coli*. The detection principle is depicted in Figure 1. Two hairpin probes, H1 and H2, were designed in this study. The target *E. coli* nucleic acid can hybridize with the toehold of H1, triggering the unwinding of the H1 hairpin structure. The loop region and the other toehold of H1 are then released and bind to the complementary sequence of H2, initiating the opening of H2 and thus activating the CHA cyclic reaction. In this process, the target molecule dissociates from the H1-H2 duplex and continues to catalyze the assembly of new H1-H2 complexes, achieving signal amplification [31]. This process simultaneously modulates two energy transfer systems. In the fluorescence detection mode, H1 is labeled with the 6-FAM fluorophore at the 5′ terminus and the BHQ-1 quencher at the 3′ terminus. In the absence of the target, both H1 and H2 exist as stable hairpin structures, and the fluorescence signal is effectively quenched via the FRET effect. As the CHA reaction proceeds, a large number of H1-H2 duplex products are generated, causing spatial separation between 6-FAM and BHQ-1, which leads to a significant fluorescence signal enhancement by disrupting the FRET effect, enabling quantitative detection of target concentration by a fluorescence spectrophotometer. In the ECL detection mode, CdS QDs are employed as the ECL signal source. H1 modified with CdS QDs is covalently immobilized onto the GCE surface via EDC/NHS coupling, while H2 is labeled with AuNPs. In the absence of the target, H1 retains its hairpin structure, and the AuNP-labeled H2 cannot approach H1, thus maintaining a stable ECL signal. Upon addition of the target, the CHA reaction is triggered, leading to the assembly of H2 with H1 on the electrode surface and bringing AuNPs into close proximity with CdS QDs. The ECL-RET effect occurs at the interface, quenching the ECL response signal. Quantitative analysis of the target molecule can thus be achieved by monitoring the ECL signal. Both detection modes are centered on CHA signal amplification, separately modulating the FRET and ECL-RET energy transfer processes to achieve synchronous output and mutual validation of the dual-modal signals, effectively enhancing the accuracy and reliability of the detection.

### 3.2. Analysis of Fluorescence Signal Response and Performance

**Figure 2:**
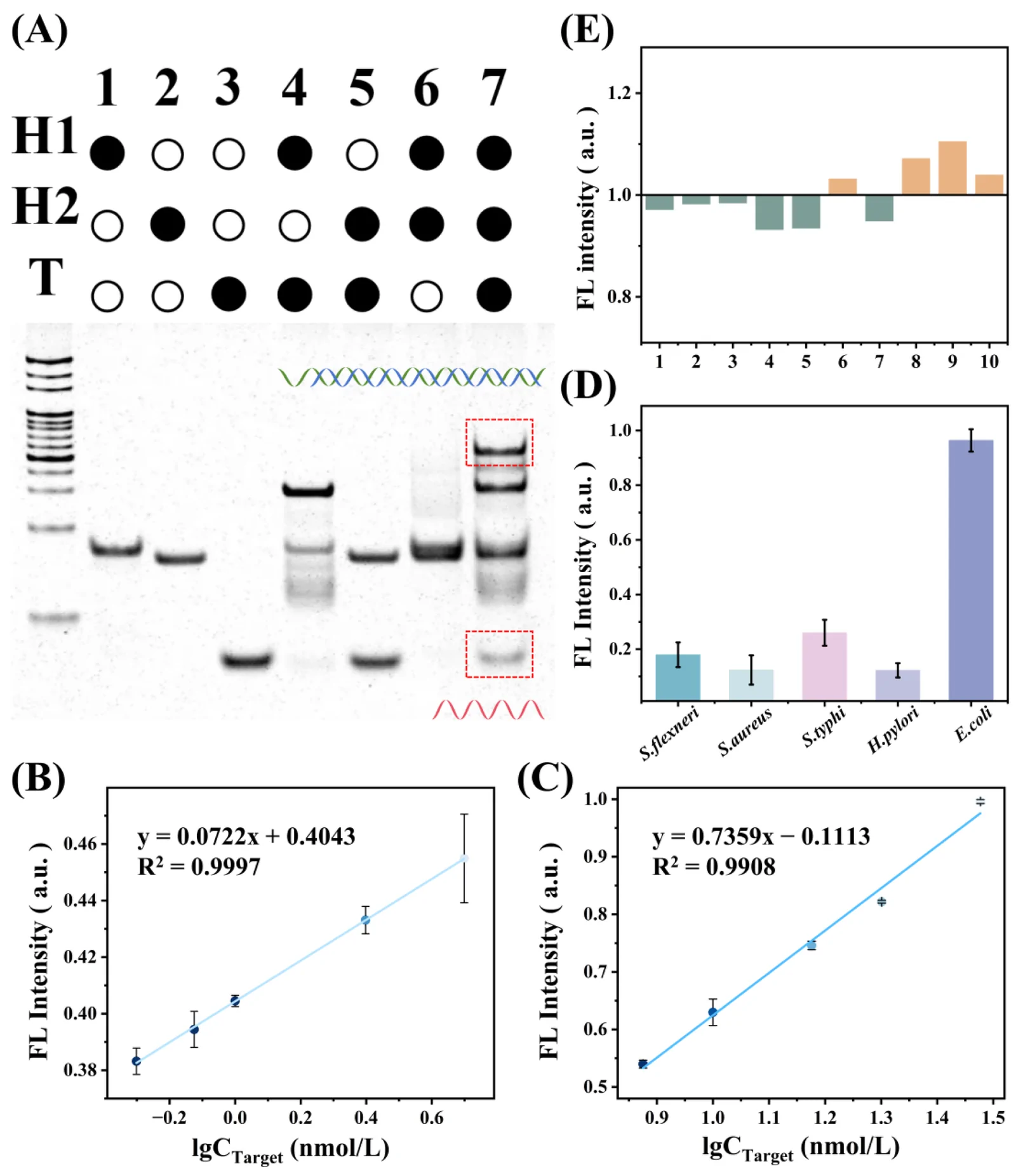
Characterization of fluorescent probe performance: (**A**) Polyacrylamide gel electrophoresis characterization of the CHA probe; (**B**) Fluorescence spectra of the *E. coli* target DNA at low concentrations; (**C**) Fluorescence spectra of the *E. coli* target DNA at high concentrations; (**D**) Evaluation of method specificity; (**E**) Evaluation of probe reproducibility.

The polyacrylamide gel electrophoresis (PAGE) characterization of the CHA reaction is shown in Figure 2A, which verified the specific response behavior of each component. As evident from the electrophoretic band distribution, lanes 1-3 correspond to the individual bands of H1, H2, and T, respectively, serving as migration references for each component. The band in lane 4 exhibited a significantly retarded migration rate compared to those of the individual H1 and T bands, confirming that probe H1 can specifically capture the target to form a binding complex. In lane 6, where the target was absent, no obvious reaction product band was observed, indicating that non-specific self-assembly between H1 and H2 was effectively suppressed. Only when H1, H2, and target were all present (lane 7) did a new high-molecular-weight complex band with a substantially slower migration rate appear, accompanied by a fading of the H1-T binding band and reappearance of the T band, indicating effective triggering of the CHA reaction. This confirms the specific recognition and signal amplification capability of the system toward the target molecule, providing a reliable experimental basis for its subsequent applications in biosensing.

In the fluorescence detection mode, the calibration curve was established using a stock solution of the target nucleic-acid sequence derived from E. coli, and the target concentration was expressed in nmol/L. Using fluorescence response signal intensity as the evaluation criterion, single-factor optimization of key reaction conditions was carried out. It was found that efficient signal amplification could be achieved when the nucleic acid strands were reacted in 1 × PBS buffer containing 0.15 mol/L NaCl (Figure S1A,B) at 37 °C for over 6 h (Figure S1C,D). Under the optimized conditions, a standard curve correlating fluorescence intensity with target concentration was systematically constructed, establishing a quantitative fluorescence detection method for the target and ensuring the accuracy and reliability of the experimental data. As shown in Figure 2B,C, the fluorescence intensity of the system exhibited a good positive linear correlation with the logarithmic value of the target concentration over both low and high concentration ranges. The fitting equation for the low concentration range was y = 0.0722x + 0.4043 (R^2^ = 0.9997), and that for the high concentration range was y = 0.7359x − 0.1113 (R^2^ = 0.9908). The limit of detection of this sensing system was calculated to be 70 pmol/L. A certain degree of variation was observed among the fluorescence measurements at different concentrations, as reflected by the error bars. Such variation is unavoidable in fluorescence-based measurements and may arise from factors associated with fluorescence signal fluctuations, sample preparation, and instrumental response. Despite the variation observed for individual measurements, the overall fluorescence response maintained a clear concentration-dependent trend, particularly in the low-concentration range, where an excellent linear correlation was obtained (R^2^ = 0.9997). These results indicate that the proposed sensing system is particularly suitable for sensitive quantitative detection at low target concentrations and may provide a useful platform for the analysis of complex real samples.

To further validate the reliability of this CHA-based detection method in microbial assays, systematic specificity and reproducibility experiments were carried out. As shown in Figure 2D, when the probes were incubated with various non-target sequences (including *S. flexneri*, *S. aureus*, *S. typhi*, and *H. pylori*), the fluorescence signals remained at relatively low levels. In contrast, upon binding to the perfectly matched target (*E. coli*), the system exhibited a significantly enhanced fluorescence signal, indicating that the probes possess strong sequence recognition specificity and can effectively distinguish the target from non-target microorganisms. Meanwhile, ten parallel samples were prepared and measured under identical experimental conditions. As shown in Figure 2E, the RSD of the fluorescence signals across all parallel samples was only 5.91%, demonstrating that the experimental procedure exhibits good operational stability and the detection results are highly reproducible and reliable.

Furthermore, to validate the applicability of the method in complex biological matrices, spike recovery experiments were conducted in a simulated biological matrix containing 2% FBS to mimic the complex environment of real biological samples, thereby providing experimental evidence for the application of the method in actual sample detection. The experimental results are summarized in Table S2. At high background concentrations (5.0 and 10.0 nmol/L), the average recoveries were 99.59% and 100.37%, with RSD values of 1.87% and 9.61%, respectively. At low background concentrations (0, 0.5, 1.0, and 2.5 nmol/L), the average recoveries ranged from 75.73% to 97.65%, with RSD values ranging from 4.33% to 10.91%. The recoveries across the entire concentration range fell within acceptable intervals: the high-concentration groups achieved accuracies approaching the ideal level, while the low-concentration groups also met the general requirements for analytical methodology. These results indicate that the detection method exhibits good accuracy and precision in complex matrices, effectively overcoming matrix interference.

### 3.3. ECL Response

**Figure 3:**
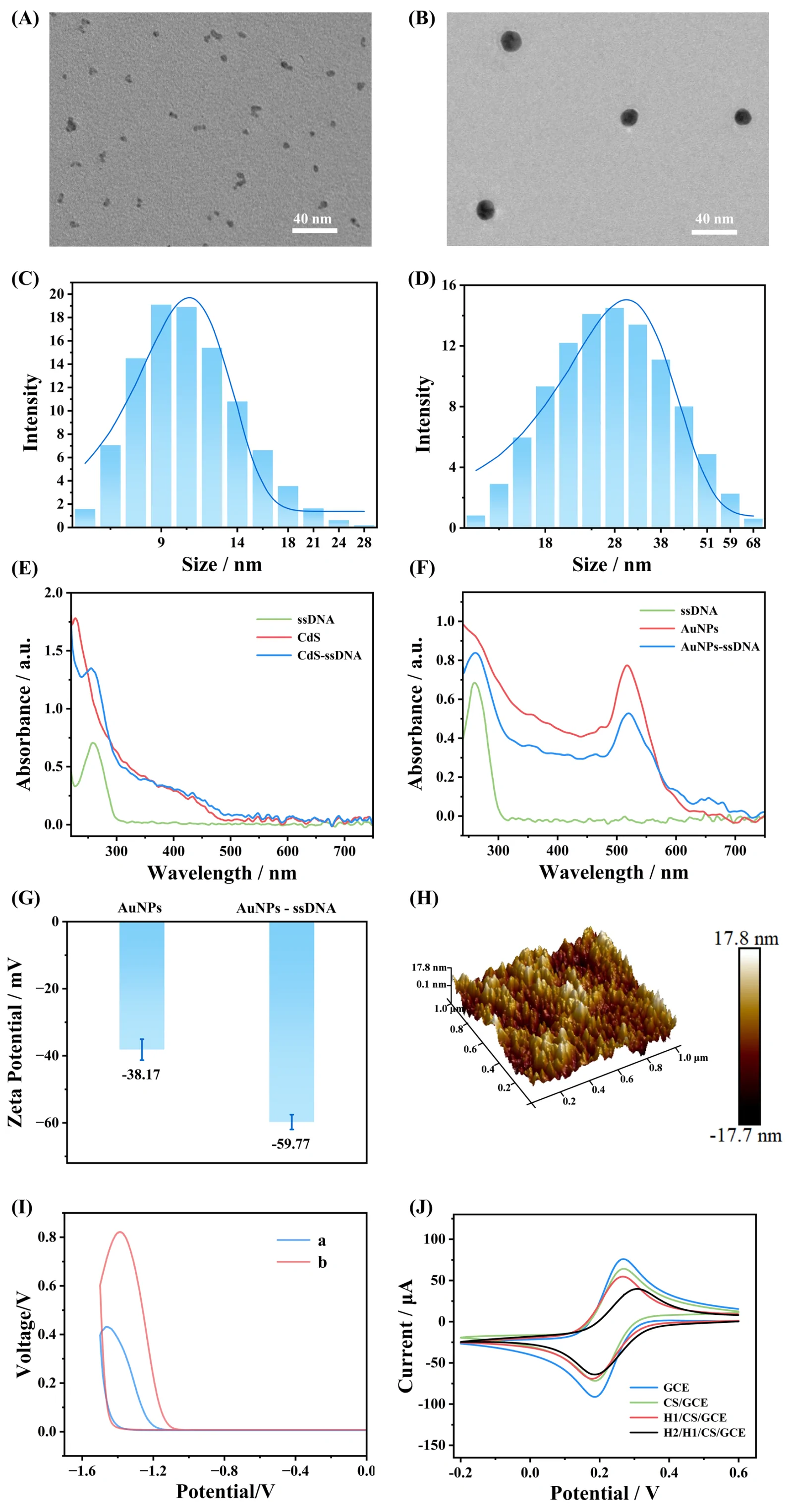
Nanomaterial and electrochemical characterizations: (**A**) Particle morphology of CdS by TEM; (**B**) Particle morphology of AuNPs by TEM; (**C**) DLS particle size distribution of CdS; (**D**) DLS particle size distribution of AuNPs; (**E**) UV-vis absorption spectrum of CdS@DNA nanoprobe; (**F**) UV-vis absorption spectrum of AuNPs@DNA nanoprobe; (**G**) Zeta potential of AuNPs@DNA nanoprobe; (**H**) Morphology of AuNPs/chitosan composite film under AFM; (**I**) Comparison of electrochemical responses of (a) the electrode modified with the chitosan film and CdS and (b) the electrode modified with the chitosan/AuNPs composite film and CdS; (**J**) Cyclic voltammetry characterization of the stepwise modification process of the sensing electrode.

The size and morphology of nanomaterials exert decisive influences on molecular recognition efficiency, signal transduction efficiency, and nucleic acid modification efficiency at the sensing interface [32,33,34]. In this study, the morphology and particle size distribution of the synthesized CdS QDs and AuNPs were characterized by transmission electron microscopy (TEM), dynamic light scattering (DLS), and fluorescence spectroscopy. As shown in Figure 3A,B, the two nanomaterials exhibited quasi-spherical and spherical structures, respectively, with small particle sizes and uniform distribution, demonstrating good monodispersity. In addition, the average hydrodynamic diameter of the CdS QDs determined by DLS was 11 nm (Figure 3C), with a narrow size distribution, indicating high purity and dimensional uniformity of the synthesized product. The average hydrodynamic diameter of the AuNPs was 32 nm (Figure 3D), with satisfactory monodispersity, meeting the size requirements for signal molecules. Both materials satisfied the requirements for the subsequent construction of the detection system, providing reliable support for their optical and electrical properties as probe signal units.

The modification efficiency of nucleic acid probes on nanomaterials is fundamental to the construction of nanosensing probes for specific target recognition [35,36]. In this study, the conjugation assembly of nucleic acid probes with the two types of nanomaterials was further verified by UV-vis absorption spectroscopy and zeta potential measurements. The results showed that bare CdS QDs and ssDNA each exhibited characteristic absorption peaks in the UV region. After covalent coupling of II-H1 onto the CdS surface via EDC/NHS activation, the absorption spectrum of CdS@DNA exhibited a similar overall trend to that of bare CdS, yet a slight peak shift was observed at specific wavelengths, indicating successful probe conjugation through covalent interactions between DNA and the CdS surface (Figure 3E). Compared with bare AuNPs, which exhibited a typical surface plasmon resonance absorption peak at approximately 520 nm, the AuNPs@DNA conjugates, formed by stable assembly of the II-H2 probe onto the AuNP surface via Au-S bonds after TCEP reduction of disulfide bonds [37], displayed a certain degree of red shift in the absorption peak with a slight change in peak shape, confirming the effective immobilization of DNA on the AuNP surface (Figure 3F). Zeta potential analysis (Figure 3G) provided direct electrical evidence for the modification process of the probe on the AuNP surface. The zeta potential of bare AuNPs was −38.17 mV. After DNA modification, the zeta potential of AuNPs@DNA decreased to −59.77 mV. This change is attributed to the abundant negatively charged phosphate groups along the DNA backbone, which significantly increased the negative charge density on the AuNP surface, shifting the potential toward more negative values [38], thereby confirming the successful modification of the DNA probe onto the AuNP surface.

Chitosan has attracted considerable attention in the construction of electrochemical sensor interfaces due to its excellent film-forming ability, good biocompatibility, and abundant chemical modification sites. The fabrication of a loose and porous chitosan modification layer is crucial for enhancing the performance of sensing electrodes [39]. The porous chitosan/AuNP composite film in our study was prepared by galvanostatic electrodeposition according to a previously reported procedure. During electrodeposition, the local pH near the cathode interface gradually increased. As the local pH approached and exceeded the pKa of chitosan, the amino groups of chitosan became progressively deprotonated, resulting in decreased solubility and subsequent precipitation of chitosan. As a result, chitosan was deposited onto the electrode surface, while AuNPs were entrapped within the polymer matrix, leading to the formation of a porous composite layer [29,40]. Atomic force microscopy (AFM) was employed to scan the prepared chitosan composite film. The morphological results (Figure 3H) revealed that the composite film surface exhibited an interlaced concave-convex porous structure with numerous depressions and pronounced surface undulations, displaying typical characteristics of porous architectures. The nanoparticles were uniformly dispersed within the chitosan matrix without obvious aggregation, indicating the successful construction of a chitosan composite film with a porous surface structure. The ECL measurements were performed in 0.1 mol/L phosphate-buffered saline (PBS, pH 7.5), following the electrochemical measurement conditions reported by Yun et al. [29]. The results are shown in Figure 3I. Compared with the electrode modified with the chitosan film and CdS, the electrode modified with the chitosan/AuNPs composite film and CdS exhibited a markedly enhanced ECL response under the same conditions. This enhancement can be attributed to the combined effects of the loose and porous architecture of the chitosan/AuNP composite film. Compared with the conventional chitosan film, the porous composite structure increased the accessible specific surface area and provided abundant active sites and favorable diffusion pathways for electroactive species, thereby facilitating interfacial mass transfer and electrochemical processes. Meanwhile, the incorporated AuNPs can facilitate interfacial electron transfer, further contributing to the enhanced electrochemical response and ECL intensity [41,42,43]. This greatly optimized the electrochemical response environment at the electrode interface, ultimately achieving a highly efficient enhancement of the ECL sensing signal.

Cyclic voltammetry (CV) was further employed to electrochemically characterize the stepwise modification process of the electrode. As shown in Figure 3J, the bare GCE exhibited a pair of typical redox peaks. After the electrode surface was modified with the chitosan composite film (CS/GCE), the redox peak currents decreased slightly. This observation indicated that the composite film had been successfully modified onto the electrode interface and impeded electron transfer at the electrode surface to a certain extent, resulting in a moderate decrease in electrode conductivity. Subsequently, the II-H1 probe was covalently coupled to the chitosan composite film layer via an EDC/NHS reaction (H1/CS/GCE), and the peak current further decreased. This can be attributed to the fact that the immobilized single-stranded DNA, with its exposed bases, assumes a flexible random-coil conformation, behaving as a typical inert insulating layer. Meanwhile, its phosphate backbone carries a large number of negative charges, which exert strong electrostatic repulsion and physical barrier effects on the negatively charged [Fe(CN)_6_]^3−^/^4−^ probe in solution, leading to a further decline in conductivity [44]. Finally, upon the addition of II-H2 and target (H2/H1/CS/GCE), the interfacial film became thicker and stiffer, and the surface negative charge density was further increased, exhibiting a stronger interfacial blocking effect compared with single-stranded DNA. Consequently, the peak currents declined continuously, the peak potential separation widened, and the reversibility deteriorated. These results collectively confirmed that each step of the electrode modification was successfully accomplished.

**Figure 4:**
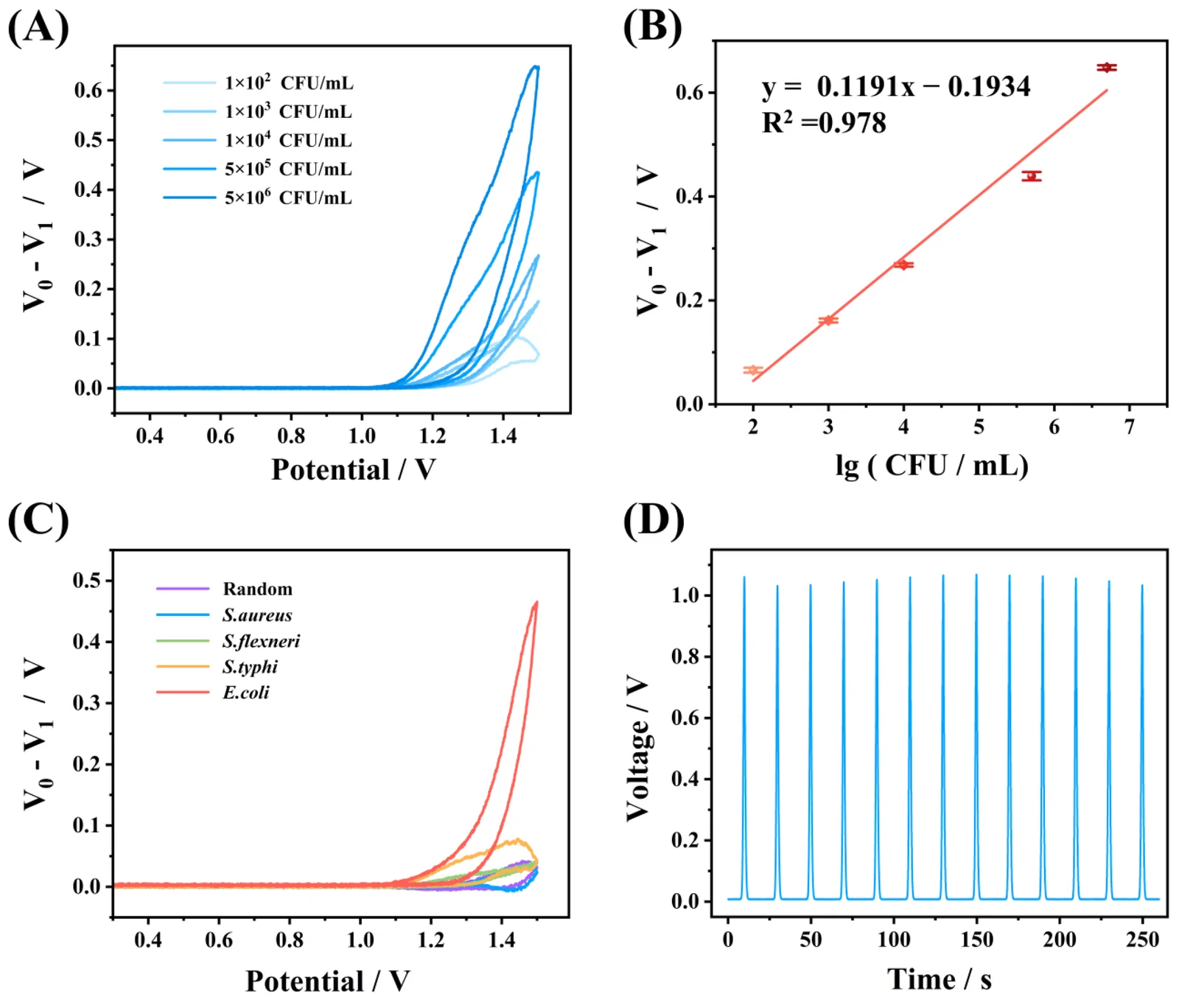
Analytical performance of the ECL biosensing platform. (**A**) ECL signals obtained with *E. coli* targets at serial concentrations ranging from 1 × 10^2^ to 5 × 10^6^ CFU/mL; (**B**) Quantitative calibration curve between potential difference and the logarithmic concentration of target; (**C**) Selectivity evaluation of the method; (**D**) Stability assessment of the ECL biosensor.

The ECL detection mode was evaluated using intact bacterial samples, and the bacterial concentration was expressed as CFU/mL. This concentration unit was used to directly represent the bacterial load in the samples and to further evaluate the applicability of the electrochemical sensing platform to bacterial detection. In ECL detection, scanning parameters, modification layer ratio, and electrode incubation time directly affect the electron transfer rate at the electrode surface and serve as critical conditions for optimizing ECL signal output [39,45,46]. In this study, the key experimental conditions for ECL detection were systematically optimized, and the optimal parameters were determined as follows: incubation time of 60 s, chitosan-to-water ratio of 2:8, and scan rate of 0.15 V s^−1^ (Figure S2A,C). Under the optimized conditions, a quantitative electrochemical detection method was established. As shown in Figure 4A, ECL signals revealed that with increasing bacterial concentration, the current signals were significantly enhanced with clear gradient discrimination. A concentration-dependent calibration curve was further constructed (Figure 4B). As the bacterial concentration gradually increased, ΔV/V_0_ exhibited an excellent linear correlation with the logarithm of bacterial concentration, with a regression equation of y = 0.1191x − 0.1934 (R^2^ = 0.978), which verified the reliable quantitative performance of this assay. The limit of quantification (LOQ) was calculated to be 100 CFU/mL based on the ECL calibration curve, which demonstrates the satisfactory quantitative performance of the ECL mode for bacterial detection. Additionally, the analytical performance of the proposed sensor was compared with other detection methods for pathogenic bacteria assays, as presented in Table S3. In the selectivity validation, the results (Figure 4C) showed that only when the target was *E. coli* did the ΔV/V_0_ value exhibit a significant increase, while the response signals for other non-target species remained near baseline levels. These results fully confirmed that the sensing system could effectively distinguish the target from non-target sequences, exhibiting high sequence recognition specificity toward the target and avoiding non-specific interference. In the stability evaluation (Figure 4D), real-time monitoring over 250 s revealed that the ECL signal peaks remained stable with consistent peak shapes and no significant attenuation or fluctuation, demonstrating excellent signal stability. Collectively, the above results demonstrated that the constructed ECL biosensor possessed outstanding quantitative detection capability, good signal stability, and high target selectivity.

### 3.4. Actual Sample Testing and Methodological Comparative Validation

To verify the accuracy and anti-interference capability of the detection system, the practical application performance of the CHA-based dual-modal detection system in complex matrices was systematically evaluated through spike recovery experiments using three typical real matrices: vegetables, water and soil samples. The results are summarized in Table S4. The spike recoveries spanned from 67.03% to 127.06%. Notably, the variation in recovery tended to decrease with increasing spiked target concentration, accompanied by improved measurement stability and repeatability. This trend may be related to the greater influence of analytical variability at low target concentrations and the differences in matrix composition and nucleic acid extraction efficiency among the tested samples. These results suggest that the proposed sensor has potential for quantitative analysis in diverse complex real samples; however, its performance may be affected under conditions involving low target concentrations and complex matrix effects. Therefore, these factors should be considered when applying the method to different real-sample matrices and practical analytical scenarios.

**Figure 5:**
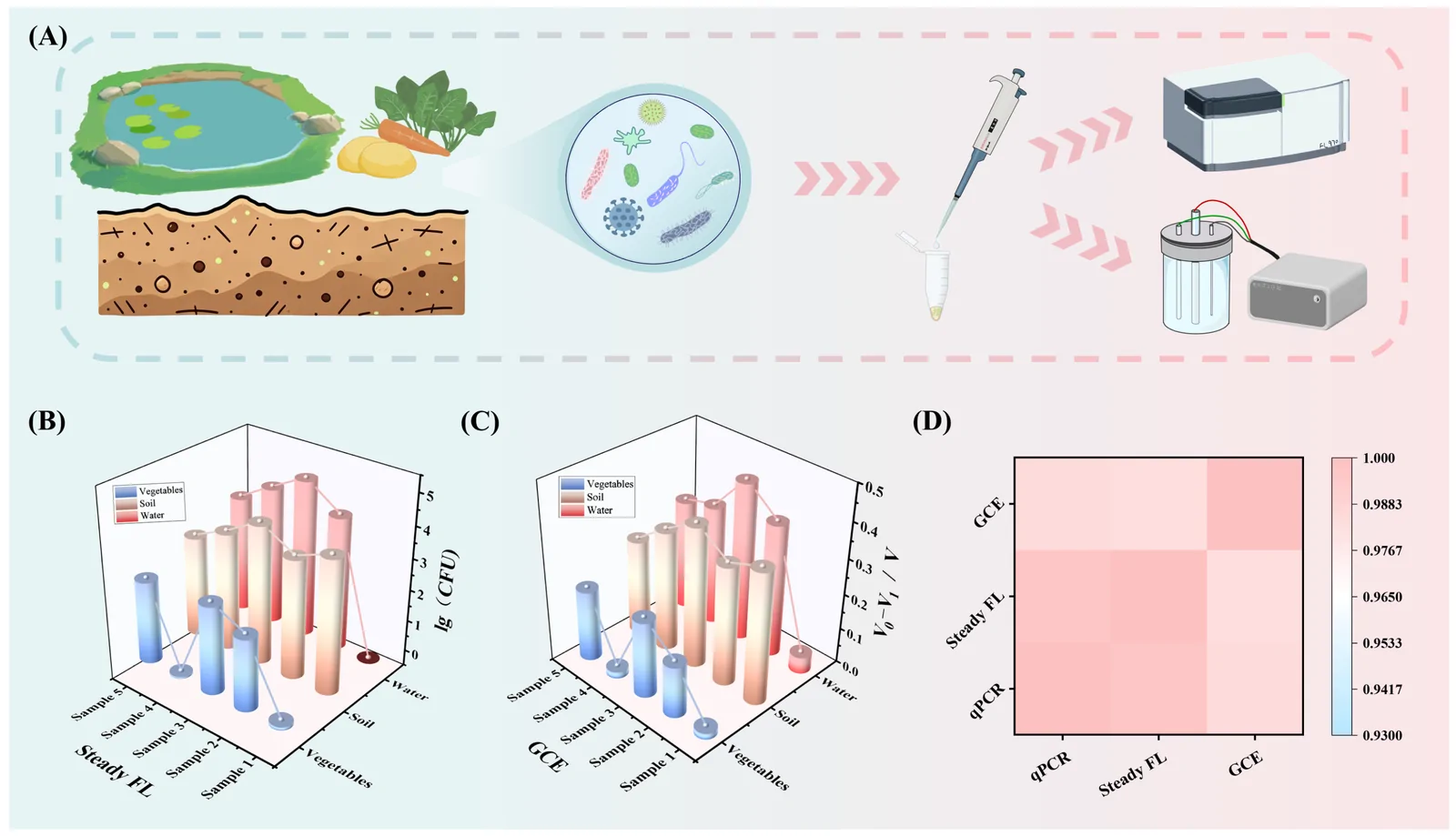
Actual sample testing: (**A**) Actual sample testing procedure; (**B**) Fluorescence detection results; (**C**) ECL detection results; (**D**) Pearson correlation analysis between the dual-modal detection system and qPCR.

To further evaluate the reliability and practical applicability of the detection system, the developed CHA-based signal amplification system was compared with the qPCR method, and its detection performance and application potential were assessed in three types of real samples: water, vegetables and soil. The detection workflow is illustrated in Figure 5A. The fluorescence detection module (Figure 5B) revealed that all three types of real matrices generated characteristic signal responses. Notably, the fluorescence signal intensities of vegetable matrices were generally lower, which may be attributed to the relatively low abundance of *E. coli* inherently present in vegetables. In contrast, the soil matrices exhibited higher signal values with smaller fluctuations, which is likely due to the abundant moisture and organic matter in soil providing a favorable growth environment for *E. coli*, resulting in higher background target concentrations. The GCE-based electrochemical detection module (Figure 5C) achieved precise discrimination of target concentrations via differential signal responses, with clearly distinguishable signal variations among different samples, and its response profiles were fully consistent with those of the fluorescence module. A single-signal detection system is susceptible to interference from matrix background, environmental impurities, and other factors, leading to uncertainty in the detection results. In contrast, when dual signals are detected simultaneously, the two detection modes can mutually corroborate each other. Only when the two signal outputs exhibit consistent trends can the detection results be deemed accurate and valid. The above results fully demonstrate that the CHA-based dual-modal biosensor constructed in this study exhibited excellent anti-interference performance, detection reproducibility, and quantitative accuracy in various complex real matrices, holding broad application prospects in the field of rapid microbial detection.

The qPCR amplification curves are shown in Figure S3. Three types of samples all exhibited typical sigmoidal amplification profiles, with the amplification onset time and fluorescence growth rate strictly corresponding to the concentration gradients of the target microorganisms in the samples. The ΔR_n_ peak values of different matrix samples were highly consistent with the signal intensities obtained from the dual-modal detection system. As a classical precise quantitative method, the qPCR results provided reliable quantitative reference standards for the dual-modal biosensor. On this basis, Pearson correlation analysis was further performed to verify the consistency among the different detection methods. As shown in Figure 5D, both fluorescence and ECL signals exhibited extremely high Pearson correlation coefficients against the qPCR results, confirming that the detection signals of the dual-modal sensing system were in excellent agreement with the quantitative data obtained by qPCR. The corresponding Pearson correlation coefficients, sample size, and statistical significance are summarized in Table S5. Collectively, these results demonstrated that the dual-modal detection system exhibited reliable quantitative accuracy in complex real matrices, which endows it with great potential for on-site rapid detection of pathogenic microbes in food and environmental matrices.

## 4. Conclusions

To address the limitations inherent in single-signal detection modes, this study successfully constructed a fluorescence-ECL dual-signal combined detection platform by integrating CHA nucleic acid amplification technology with the signal modulation properties of CdS QDs and AuNPs, using *E. coli* as the detection target. Through optimization of the CHA amplification reaction, nanoprobe assembly, and key ECL detection conditions, highly sensitive and specific detection of the target nucleic acid was achieved. The fluorescence module exhibited a good linear relationship over a certain concentration range with a detection limit as low as 70 pmol/L, demonstrating a broad linear detection range, ultra-high sensitivity, and accurate quantitative performance. The ECL module exhibited a favorable linear response over a specific concentration range, along with excellent signal stability and target recognition specificity. Detection results in real sample matrices revealed that the two detection modes yielded highly consistent outputs under complex sample backgrounds without observable mutual signal interference, indicating that this dual-modal detection system can effectively overcome environmental background interference and hold potential application value in the rapid and accurate detection of pathogenic microorganisms.

## Data Availability

The original contributions presented in the study are included in the article’s Supplementary Materials. Further inquiries can be directed to the corresponding author.

## Supplementary Materials

The following supporting information can be downloaded at: https://www.mdpi.com/article/10.3390/bios16100556/s1, Figure S1: Optimization of the conditions for the CHA reaction; Figure S2: Optimization of key experimental conditions for ECL detection; Figure S3: qPCR amplification curves in real samples; Table S1: Oligonucleotide sequences used in this study; Table S2: Recycling efficiency results for the Target in a 2% FBS solution; Table S3: Comparison of the analytical performance of the proposed dual-modal CHA biosensor with representative single-modal CHA and dual-modal pathogen; Table S4: Recovery results from spiked recovery experiments conducted in various actual matrices; Table S5: Pearson correlation analysis of the Steady FL, GCE, and qPCR results. Refs. [22,47,48] cited in Supplementary Materials.

## References

[1] Yu Q., Wu T., Tian B., Li J., Liu Y., Wu Z., Jin X., Wang C., Wang C., Gu B. (2024). Recent advances in SERS-based immunochromatographic assay for pathogenic microorganism diagnosis: A review. Anal. Chim. Acta.

[2] Lanzas C., Davies K., Erwin S., Dawson D. (2019). On modelling environmentally transmitted pathogens. Interface Focus.

[3] Balloux F., van Dorp L. (2017). Q&A: What are pathogens, and what have they done to and for us?. BMC Biol..

[4] Albalawi S.S., Akhtar N., El-Said W.A. (2025). Real-Time and Selective Detection of Pseudomonas aeruginosa in Beef Samples Using a g-C3N4-Doped Multimetallic Perovskite-Based Electrochemical Aptasensor. Biosensors.

[5] Chen F., Li G.D., Wu C., Wang W.H., Ma D.L., Leung C.H. (2022). A rapid and label-free DNA-based interference reduction nucleic acid amplification strategy for viral RNA detection. Biosens. Bioelectron..

[6] Lin G., Wang Z., Li Q., Zhang J., Guo S., Xu H., Lin S., Xie X. (2025). Confined CHA-HCR system for sensitive and specific detection of ANXA2 mRNA in adenomyosis tissues. J. Pharm. Biomed. Anal..

[7] Mo L., He W., Li Z., Liang D., Qin R., Mo M., Yang C., Lin W. (2023). Recent progress in the development of DNA-based biosensors integrated with hybridization chain reaction or catalytic hairpin assembly. Front. Chem..

[8] Liu Z., Liu C., He L., Liu J., Li L., Yang S., Tan Y., Liu X., Xiao X. (2023). A cascade signal amplification strategy for the ultrasensitive fluorescence detection of Cu^2+^ via λ-exonuclease-assisted target recycling with mismatched catalytic hairpin assembly. Biosensors.

[9] Zhao L.Y., Song Y., Xu H.Y. (2024). Catalytic hairpin self-assembly for biosensing: Classification, influencing factors, and applications. TrAC Trends Anal. Chem..

[10] Raina M.B., Jesse V.J. (2021). Hybridizing clinical translatability with enzyme-free DNA signal amplifiers: Recent advances in nucleic acid detection and imaging. Biomater. Sci..

[11] Shi K., Chen J.X., Song J.H., Yuan R., Xiang Y. (2025). Target-promoted formation of DNA four-way junction for catalytic hairpin assembly amplification and electrochemical SNP assay in folded hairpin structure. Sens. Actuators B.

[12] Xu Y., Li L., Yang Y., Huang C., Zou H. (2024). Catalytic hairpin assembly triggering amplified DNAzyme-feedback for sensitive detection of hepatitis C virus genotype 1b. Talanta.

[13] Sun X., Zhang Y., Lu X., Xu H., Yuan Y., Wang M., Zhao Y., Zhang W. (2025). Research progress of enzyme-free DNA self-assembly technology in rapid detection of food safety. J. Food Compos. Anal..

[14] Zhong L.M., Li C.M., Zhang J., Li Y.X., Zhuang J., Zhong X.Y., Kong F.Y., Hu S.W. (2026). Dual Confinement-Enhanced Multiple Single Nucleotide Variant Detection at the Single-Particle Level. ACS Sens..

[15] Dong J., Lu T., Zhang Y., Su M., Wang J., Fan Y., Li Y., Yan H., Shen S., Ma Q. (2025). Aptamer-anchored hydrophobic nanopores for electrochemical/fluorescent dual-mode identification of pathogenic bacteria. Chem. Eng. J..

[16] Gil B., Keshavarz M., Wales D., Darzi A., Yeatman E. (2023). Orthogonal surface-enhanced raman scattering/field-effect transistor detection of breast and colorectal cancer-derived exosomes using graphene as a tag-free diagnostic template. Adv. NanoBiomed Res..

[17] Cai J., Lin Y., Yu X., Yang Y., Hu Y., Gao L., Xiao H., Du J., Wang H., Zhong X. (2023). Multifunctional AuAg-doping Prussian Blue-based MOF: Enhanced colorimetric catalytic activities and amplified SERS signals for bacteria discrimination and detection. Sens. Actuators B.

[18] Esmaelpourfarkhani M., Ramezani R., Alibolandi M., Abnous K., Taghdisi S.M. (2023). Time-resolved fluorescence DNA-based sensors for reducing background fluorescence of environment. J. Fluoresc..

[19] Zhang L., Ismail N.I. (2026). Nanomaterials-based electrochemical aptasensors for rapid detection of pathogens and by-products. Molecules.

[20] Guo W., Jiang M., Xie Y., Xu H., Sun Z. (2025). Recognition Element-Based Strategies for Rapid Detection of Foodborne Pathogens: Recent Progress and Perspectives. Biosensors.

[21] Gao H., Zhang H., Qi X., Miao M., Que L., Gu X., Chang D., Pan H. (2025). CRISPR/Cas12a dual-mode biosensor for *Staphylococcus aureus* detection via enzyme-free isothermal amplification. Talanta.

[22] Feng Q., Wu T., Wang H., Wu M., Dou B., Wang P. (2024). Two-step resonance-energy-transfer-based ratiometric biosensor for sensing and annihilation of *Staphylococcus aureus*. Chem. Commun..

[23] Zhang N., Liu X., Wei Z., Liu H., Peng J., Zhou L., Li H., Fan H. (2019). Cell imaging using two-photon excited CdS fluorescent quantum dots working within the biological window. Nanomaterials.

[24] Doyen M., Bartik K., Bruylants G. (2013). UV-Vis and NMR study of the formation of gold nanoparticles by citrate reduction: Observation of gold-citrate aggregates. J. Colloid Interface Sci..

[25] Wuithschick M., Birnbaum A., Witte S., Sztucki M., Vainio U., Pinna N., Rademann K., Emmerling F., Kraehnert R., Polte J. (2015). Turkevich in new robes: Key questions answered for the most common gold nanoparticle synthesis. Acs Nano.

[26] Liu J., Lu Y. (2006). Preparation of aptamer-linked gold nanoparticle purple aggregates for colorimetric sensing of analytes. Nat. Protoc..

[27] Li P., Luo C., Chen X., Huang C. (2022). An off-on fluorescence aptasensor for trace thrombin detection based on FRET between CdS QDs and AuNPs. RSC Adv..

[28] Hu S.W., Zhong X.Y., Deng Y., Deng Y.H., Chen L.L. (2024). An initial check-reexamination strategy for analysis of *H. Pylori* DNA and single-nucleotide variants. Sens. Actuators B.

[29] Yun W., Xu Y., Dong P., Ma X., He P., Fang Y. (2009). Solid-state electrochemiluminescence sensor through the electrodeposition of Ru(bpy)32+/AuNPs/chitosan composite film onto electrode. Anal. Chim. Acta.

[30] Yin P., Choi H.M.T., Calvert C.R., Pierce N.A. (2008). Programming biomolecular self-assembly pathways. Nature.

[31] Shi X., Zhang Y., Sun Y., Ge C., Zhang R., Jia R., Liu Z., Zhang S., Song X. (2026). Catalytic hairpin assembly-driven DNAzyme for sensitive point-of-care colorimetric detection of *Escherichia coli* O157:H7. Sens. Actuators B.

[32] Chang H.X., Wang Y., Li J.H. (2011). Electrochemical DNA sensors: From nanoconstruction to biosensing. Curr. Org. Chem..

[33] Saha K., Agasti S.S., Kim C., Li X.N., Rotello V.M. (2012). Gold nanoparticles in chemical and biological sensing. Chem. Rev..

[34] Zhai C., Zhang H., Du N., Chen B., Huang H., Wu Y., Yang D. (2011). One-pot synthesis of biocompatible CdSe/CdS quantum dots and their applications as fluorescent biological labels. Nanoscale Res. Lett..

[35] Alex S., Tiwari A. (2015). Functionalized gold nanoparticles: Synthesis, properties and applications-a review. J. Nanosci. Nanotechnol..

[36] Cutler J.I., Auyeung E., Mirkin C.A. (2012). Spherical nucleic acids. J. Am. Chem. Soc..

[37] Liu B., Liu J. (2019). Interface-driven hybrid materials based on DNA-functionalized gold nanoparticles. Matter.

[38] Wang S.H., Lee C.W., Chiou A., Wei P.K. (2010). Size-dependent endocytosis of gold nanoparticles studied by three-dimensional mapping of plasmonic scattering images. J. Nanobiotechnol..

[39] Smułka A., Cieślik M., Olejnik A., Zieliński A., Ryl J., Ossowski T. (2025). Unlocking the electrochemical performance of glassy carbon electrodes by surface engineered, sustainable chitosan membranes. Bioelectrochemistry.

[40] Sorlier P., Denuzière A., Viton C., Domard A. (2001). Relation between the degree of acetylation and the electrostatic properties of chitin and chitosan. Biomacromolecules.

[41] Liu J., Wang X., Wang T., Li D., Xi F., Wang J., Wang E. (2014). Functionalization of monolithic and porous three-dimensional graphene by one-step chitosan electrodeposition for enzymatic biosensor. ACS Appl. Mater. Interfaces.

[42] Qiu J.-D., Wang R., Liang R.-P., Xia X.-H. (2009). Electrochemically deposited nanocomposite film of CS-Fc/Au NPs/GOx for glucose biosensor application. Biosens. Bioelectron..

[43] Xiang C., Zou Y., Sun L., Xu F. (2007). Direct electrochemistry and electrocatalysis of cytochrome c immobilized on gold nanoparticles-chitosan-carbon nanotubes-modified electrode. Talanta.

[44] Naghshgar N., Hosseinzadeh S., Derakhshandeh A., Shaali R., Doroodmand M.M. (2024). Introducing a portable electrochemical biosensor for *Mycobacterium avium* subsp. paratuberculosis detection using graphene oxide and chitosan. Sci. Rep..

[45] Zhang W., Xiong H., Chen M., Zhang X., Wang S. (2017). Surface-enhanced molecularly imprinted electrochemiluminescence sensor based on Ru@SiO_2_ for ultrasensitive detection of fumonisin B1. Biosens. Bioelectron..

[46] Pang X., Li J., Zhao Y., Wu D., Zhang Y., Du B., Ma H., Wei Q. (2015). Label-free electrochemiluminescent immunosensor for detection of carcinoembryonic antigen based on nanocomposites of GO/MWCNTs-COOH/Au@CeO_2_. ACS Appl. Mater. Interfaces.

[47] Han J., Bu S., Zhou H., Hao Z., Duan M., Li Z., Wan J. (2024). A novel fluorescent biosensor for sensitive detection of pathogenic bacteria based on catalytic hairpin assembly and λ-Exonuclease cleavage reaction. Sens. Actuators B.

[48] Liu D., Lv X., Zhao C., Li J., Huang J., Weng L., He L., Liu S. (2022). NaBiF4 upconversion nanoparticle-based electrochemiluminescent biosensor for *E. coli* O157 : H7 detection. RSC Adv..

