# Supplementary material for: A Porous Chitosan Film-Based Dual-Modal Biosensor Integrated with Catalytic Hairpin Assembly for Sensitive Detection of Escherichia coli
Source: Biosensors (Basel). 2026 Oct 2;16(10):556. doi: 10.3390/bios16100556 (PMC13702713; doi:10.3390/bios16100556)
Supplement: Supplementary file 1 [file biosensors-16-00556-s001.zip › biosensors-4584858-supplementary.pdf]

A Porous Chitosan film-Based Dual-Modal Biosensor Integrated with Catalytic Hairpin Assembly for Sensitive Detection of *Escherichia coli*

Yuhuan Wei [1], Qibin Wu [1], Hui Zhang [1], Jiayao Lin [1], Zhen Su [1], Sirui Yi [1],Kang Yang[1], Xiaomeng Jia [2], and Shanwen Hu [1,*]

Table S1. Oligonucleotide sequences used in this study
Table S2. Recycling efficiency results for the Target in a 2% FBS solution
Table S3. Comparison of the analytical performance of the proposed dual-modal CHA biosensor with representative single-modal CHA and dual-modal pathogen
Table S4. Recovery results from spiked recovery experiments conducted in various actual matrices
Table S5. Pearson correlation analysis of the Steady FL, GCE, and qPCR results
Figure S1. Optimization of the conditions for the CHA reaction
Figure S2. Optimization of key experimental conditions for ECL detection
Figure S3. qPCR amplification curves in real samples

Table S1. Oligonucleotide sequences used in this study

Name	Sequences（5’-3’）
E.coil DNA	GGTACATATTCATGGTATTCAGA
I-H1	6-FAM-CGATTATATTCATGGTATTCCCTTTAACAGCG CATGTTCTGAATACCATGAATATGTAC-BHQ-1
I-H2	TTTAACAGCGCATCATATTCATGGTATTCAGACATG CGCTGTTAAAG GAATAAAAAA
II-H1	CGATTATATTCATGGTATTCCCTTTAACAGCGCATGTT CTGAATACCATGAATATGTAC-NH2
II-H2	HS-SH-AAAAAATTTAACAGCGCATCATATTCATGGT ATTCAGACATGCGCTGTTAAAG GAATAA
S.aureus DNA	CGGACGAGAAGCTTGCTTCTCT
S. typhi DNA	GGCGGCGGGCGTCGCGCACG

S. flexneri DNA	CCGCAGCACGTTCTTGCTTATGCA
H. pylori	TTCCTCCTACCCGCGGCAAGACGGAAAGAC
Random	GCAAGCGGTGGTGATGGTACA
Forward primer	AGTTTGATCMTGGCTCAG
Reverse primer	GGTTACCTTGTTACGACTT

Table S2. Recycling efficiency results for the Target in a 2% FBS solution

Sample	Background concentration (nmol/L)	Add (nmol/L)	Found (nmol/L)	Average recovery(%)	RSD (%)
1		0.5	0.42		
2	0	0.5	0.52	94.23	10.91
3		0.5	0.48		
4		0.5	1.02		
5	0.5	0.5	0.94	97.65	4.33
6		0.5	0.97		
7		1.5	2.01		
8	1	1.5	1.92	75.73	7.18
9		1.5	1.75		
10		2.5	4.15		
11	2.5	2.5	4.33	88.55	7.56
12		2.5	4.80		
13		5	9.97		
14	5	5	9.77	99.59	1.87
15		5	10.14		
16		5	16.20		
17	10	5	15.53	100.37	9.61
18		5	13.43		

Table S3. Comparison of the analytical performance of the proposed dual-modal CHA biosensor with representative single-modal CHA and dual-modal pathogen biosensors.

**Category**	**Amplification strategy**	LOD	Linear range	**Assay time**	**matrix tolerance**	**Ref.**
Single-modal CHA fluorescence sensors	CHA + λ-exonuclease	10 CFU/mL	$10–10^8$ CFU/mL	NR	No nucleic-acid extraction; complex-matrix validation not clearly reported	47
Single-modal ECL sensors	AuNP/UCNP-assisted ECL signal amplification	138 CFU/mL	$200\text{-}10^5$ CFU/mL	45 min	Food samples	48
Dual-modal, non-porous scaffold	Dual RET + CHA	1 CFU/mL	$0\text{-}10^8$ CFU/mL	NR	Food samples	22
Present work with porous scaffold	Dual RET + CHA	70 pmol L^{-1}/ 100 CFU mL^{-1}	1×10^2 to 5×10^6 CFU/mL	6 h	Soil, water and vegetables	/

Table S4. Recovery results from spiked recovery experiments conducted in various actual matrices

Numble	Sample Type	Add (CFU/mL)	Found (CFU/mL)	Recovery (%)	Average recovery(%)	RSD (%)
	Vegetables		96	96.47		
1	Soil	1×10^{2}	71	70.98	89.79	16.52
	Water		102	101.93		
	Vegetables		3.35×10^{3}	67.03		
2	Soil	5×10^{3}	3.98×10^{3}	79.69	75.68	9.91
	Water		4.02×10^{3}	80.32		
	Vegetables		1.12×10^{4}	78.66		
3	Soil	1.5×10^{4}	1.10×10^{4}	73.28	73.05	19.15
	Water		1.01×10^{4}	67.21		
	Vegetables		5.36×10^{4}	105.19		
4	Soil	5×10^{4}	6.35×10^{4}	127.06	107.14	7.84
	Water		4.46×10^{4}	89.17		
	Vegetables		1.38×10^{5}	92.28		
5	Soil	1.5×10^{5}	1.21×10^{5}	80.73	83.95	8.67
	Water		1.18×10^{5}	78.85		
	Vegetables		2.10×10^{5}	105.01		
6	Soil	2×10^{5}	2.08×10^{5}	104.19	94.69	18.13
	Water		1.50×10^{5}	74.88		
	Vegetables		7.81×10^{5}	104.18		
7	Soil	7.5×10^{5}	7.45×10^{5}	99.37	98.71	5.90
	Water		6.94×10^{5}	92.58		
	Vegetables		5.20×10^{6}	104.04		
8	Soil	5×10^{6}	5.20×10^{6}	104.04	99.94	7.11
	Water		4.59×10^{6}	91.74		

Table S5. Pearson correlation analysis of the Steady FL, GCE, and qPCR results

**Comparison**	**Pearson's *r***	***p***	***n***
qPCR vs. Steady FL	0.998**	<0.001	15
qPCR vs. GCE	0.985**	<0.001	15
Steady FL vs. GCE	0.984**	<0.001	15

Note: **p < 0.01.

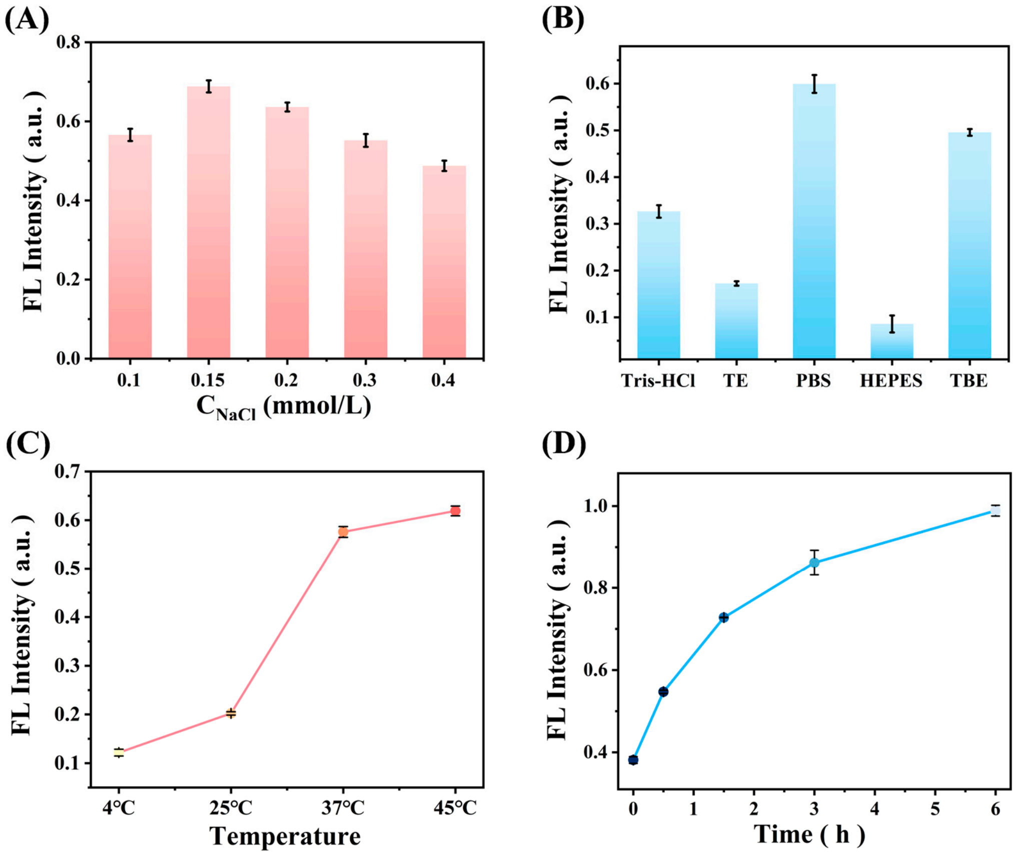

Figure S1. Optimization of the conditions for the CHA reaction. (A) Fluorescence intensity of the reaction at different NaCl concentrations; (B) Fluorescence intensity of the reaction under different buffer systems; (C) Fluorescence intensity of the reaction at different temperatures; (D) Fluorescence kinetic characteristics of the CHA reaction.

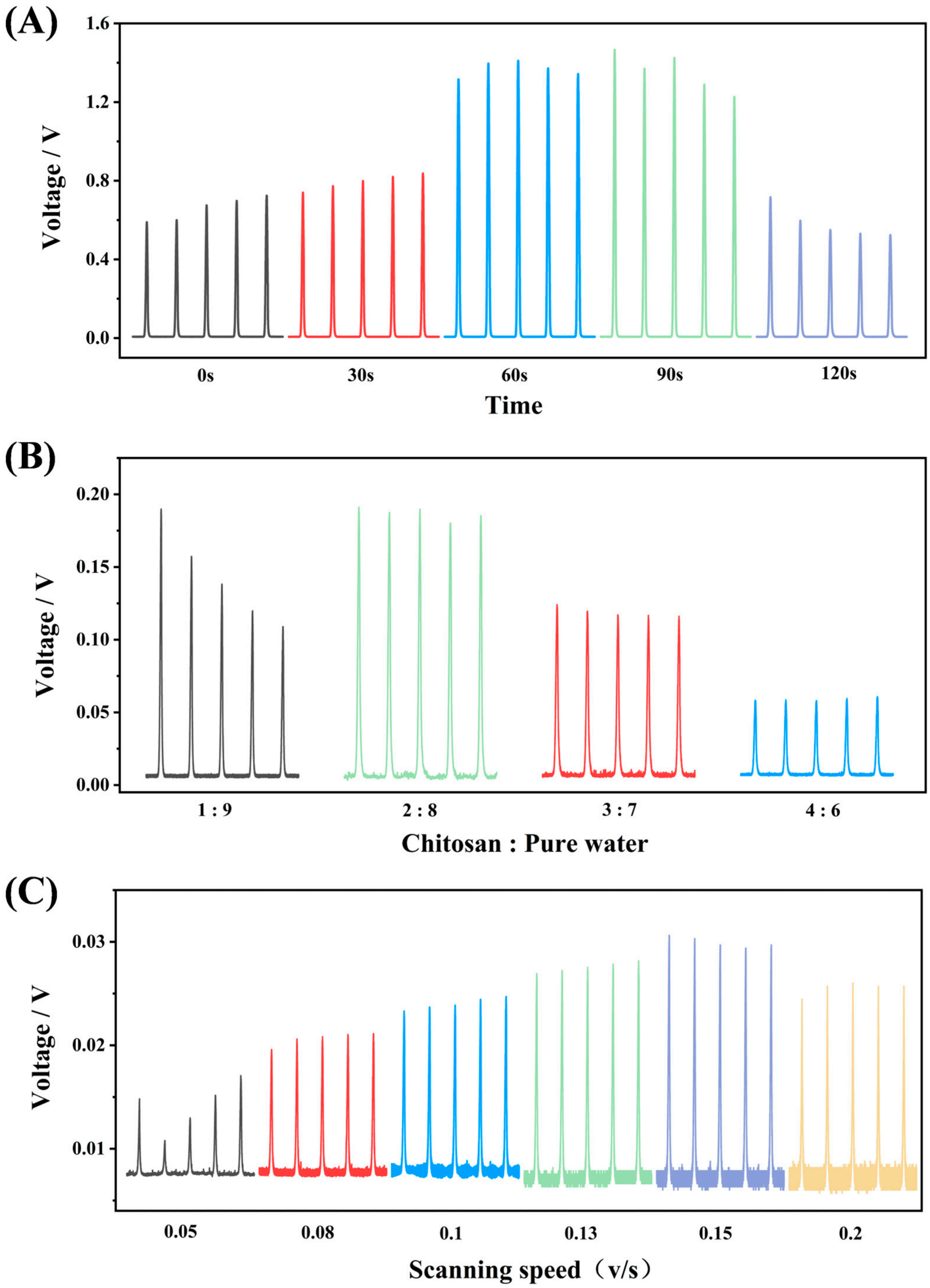

Figure S2. Optimization of key experimental conditions for ECL detection. (A) Effect of static incubation time on the ECL signal; (B) Effect of the chitosan-pure water ratio on the ECL signal; (C) Effect of the scanning speed on the ECL signal.

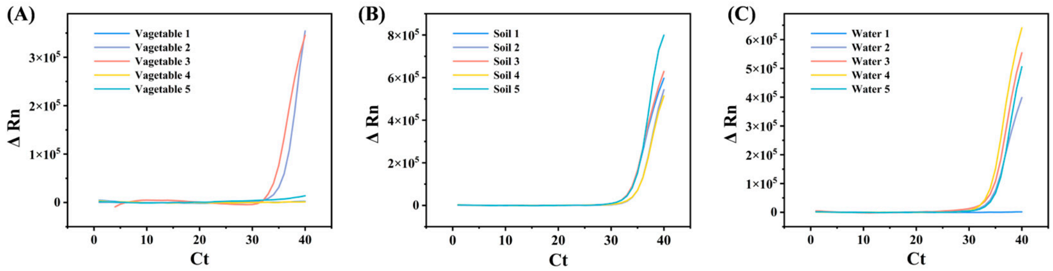

Figure S3. qPCR amplification curves in real samples. (A) qPCR amplification curves of vegetable samples; (B) qPCR amplification curves of soil samples; (C) qPCR amplification curves of water samples.
